# Improved interoceptive awareness in professional performers of the traditional Japanese theatrical art “Noh”

**DOI:** 10.3389/fpsyg.2026.1818785

**Published:** 2026-07-13

**Authors:** Hikaru Koike, Michio Nomura

**Affiliations:** Graduate School of Education, Kyoto University, Kyoto, Japan

**Keywords:** interoception, interoceptive awareness, Noh, physical sensation, theatrical artist

## Abstract

“Noh” is a traditional Japanese theatrical art characterized by sophisticated emotional expression. Noh is performed by experts in various roles such as shite-kata (actors) and hayashi-kata (instrumentalists). Although previous studies have suggested that Noh performers acquire distinctive physical techniques, including specific breathing patterns during expression, it remains unclear whether practicing Noh also leads to psychological changes. In this study, we examined trait-level changes in interoceptive awareness that accompany the practice of Noh. In the survey, 22 professional Noh performers (10 actors, *M*_age_ = 45.60, *SD*_age_ = 12.29, age range = 23–56; 12 instrumentalists, *M*_age_ = 47.50, *SD*_age_ = 10.85, age range = 25–62; all male) and 18 controls with no Noh experience (*M*_age_ = 40.50, *SD*_age_ = 6.85, age range = 31–52; all male) completed a series of questionnaires, including psychological scales on multiple aspects of interoceptive awareness, such as beliefs about interoceptive accuracy or attention. The actors showed significantly higher scores than the controls on measures of belief in interoceptive attention. On the other hand, no significant correlations between interoceptive awareness and years of Noh experience remained after correcting for multiple comparisons. These findings provide important insights into the physical and psychological techniques of Noh performers and contributes to the elucidation of factors that alter interoceptive awareness over time.

## 1 Introduction

“Noh” is a representative Japanese theatrical art established in the fourteenth century. Characterized by sophisticated emotional expression through singing, dance, and instrumental music, Noh has been the subject of research from various perspectives, including its musical characteristics (Malm, 1975) and the recognition of facial expression in masks that are used in Noh (Minoshita et al., 1999; Miyata et al., 2012). Empirical studies on the Noh performers have revealed that they show distinctive breathing patterns (Kobayashi and Morishita, 2000). Although changes in performers’ facial expression were suppressed during performance, emotional states are conveyed through changes in breathing (Homma et al., 2006). These findings are considered to be a manifestation of the physical techniques that were acquired in the process of achieving the ideal performance as a Noh performer. Th present study examined how expertise in Noh influences interoception, a process that links the body to the mind.

Interoception is a general term that refers to the processes of perceiving, interpreting, and integrating signals that arise from within the body (Craig, 2002; Desmedt et al., 2023). In recent studies, sensations regarding various domains below the skin, such as heartbeats, pain, and tension of skeletal muscles are described as interoception (Crucianelli and Ehrsson, 2023; Ceunen et al., 2016; Murphy et al., 2018). In particular, aspects that are at the level of consciousness are often referred to as interoceptive awareness (Khalsa et al., 2018; Mehling et al., 2012). Interoceptive awareness is typically treated as a dispositional trait and is commonly measured by behavioral tasks, self-report questionnaires, or composite indices derived from these measures (Garfinkel et al., 2015). Although the term “interoceptive awareness” is sometimes used more narrowly to refer only to such composite indices (Garfinkel et al., 2015), in the present study it refers more broadly to all consciously accessible aspects of interoception. More recently, indices that rely particularly on self-report, such as questionnaire scores, have also been termed interoceptive sensibility (Garfinkel et al., 2015). Both classical and contemporary theories of emotion have emphasized the central role of interoception in emotional experience (Barrett, 2017; James, 1890), and associations between these processes have been repeatedly reported across multiple levels of analysis, ranging from behavioral and self-report measures (Schandry, 1981; Wiens et al., 2000; Ventura-Bort et al., 2021) to neural activity (Adolfi et al., 2017; Terasawa et al., 2013). Furthermore, It has been argued that interoception, as a source of emotional experience, can be the basis for diverse psychological functions, including intuitive decision-making (Dunn et al., 2010), emotion regulation (Füstös et al., 2013; Pollatos et al., 2015), the perception of others’ emotions (Ondobaka et al., 2017; Shah et al., 2017), and mindfulness (Holzel et al., 2011).

Many researchers have argued that interoceptive awareness is fundamentally linked to the processes necessary for an adaptive social life. Based on this view, it is possible that improving interoceptive awareness may lead to emotional and cognitive changes. Some studies have suggested that certain aspects of interoception are associated with maladaptive emotion such as increased anxiety (Critchley et al., 2004), which can also be tested by interventions that alter interoception over time. However, the factors that contribute to long-term changes in interoceptive awareness remain poorly understood. To address this issue, several studies have examined interoceptive awareness in experts from various disciplines and compared them with individuals with no such experience. Previous researches have shown that artists including singers, stringed instrumentalists (Schirmer-Mokwa et al., 2015), and ballet dancers (Christensen et al., 2018) exhibit high levels of interoceptive awareness. These studies reported that artists can perceive their heartbeats more accurately than non-artists, and that heartbeat perception accuracy is positively correlated with years of artistic experiences. Such findings support the dual-action hypothesis (Christensen et al., 2018), which proposes that two activities of professional artistic training enhance interoceptive awareness. Two activities proposed in this hypothesis are as follows: (1) eliciting physical states by remembering autobiographical memories, and (2) immediately expressing those physical states. At a fine-grained level, “eliciting physical states” involves the process of paying attention to the body (see Christensen et al., 2018). However, these studies (Schirmer-Mokwa et al., 2015; Christensen et al., 2018) assessed interoceptive awareness solely through heartbeat perception accuracy (Schandry, 1981; Brener and Kluvitse, 1988). Examining dimensions of interoceptive awareness beyond heartbeat perception accuracy may provide findings that complement and extend the dual-action hypothesis.

A recent model (Murphy et al., 2018) organizes measures of interoceptive awareness into 2 × 2 factors based on the dimensions of measured object (accuracy or attention) and measurement method (task performance or self-reported beliefs). Interoceptive attention refers to the ability to direct attention toward bodily signals or to beliefs about the extent to which one pays attention to one’s body (Desmedt et al., 2023; Gabriele et al., 2022; Khalsa et al., 2018). Accuracy-related variables, whether assessed through self-report or behavioral performance, tend to be positively correlated one another (Garfinkel et al., 2015; Koike and Nomura, 2023, 2026; Murphy et al., 2020). In contrast, accuracy-related and attention-related variables are rarely correlated and exhibit different patterns of association with other psychological variables (Koike and Nomura, 2023, 2026; Murphy et al., 2020; Gabriele et al., 2022). Therefore, from both basic and applied research perspectives, it is important to examine the effects of artistic practice on each aspect of interoceptive awareness. In particular, questionnaire-based measures make it possible to assess sensibility to signals arising from various regions of the body, rather than focusing exclusively on heartbeat perceptions.

In the present study, three psychological scales were used to assess interoceptive awareness. The Interoceptive Accuracy Scale (IAS; Murphy et al., 2020; Koike and Nomura, 2023) measures beliefs regarding accuracy within the 2 × 2 model (Murphy et al., 2018). The Interoceptive Attention Scale (IATS; Gabriele et al., 2022; Koike and Nomura, 2023) measures beliefs regarding attention within the same 2 × 2 model. The Multidimensional Assessment of Interoceptive Awareness (MAIA; Mehling et al., 2012; Shoji et al., 2018) measures broader aspects of interoceptive awareness, such as the extent to which individuals trust their own bodily sensations, although some of its subscales correspond to beliefs regarding interoceptive attention. The MAIA was included as a complementary measure to capture aspects of interoception that are not fully represented for within the 2 × 2 factors. According to the dual-action hypothesis (Christensen et al., 2018), the first stage of emotional expression involves eliciting bodily signals and directing attention toward them. Therefore, improvements in interoceptive attention may precede improvements in interoceptive accuracy. Consequently, expertise in Noh may be associated not only with changes in accuracy-related variable but also with changes in attention-related variables.

Another unexplored issue is that, although the dual-action hypothesis (Christensen et al., 2018) provides a theoretical account of the relationship between artistic practice and interoceptive awareness, empirical evidence supporting its underlying mechanism remains limited. Previous studies have primarily demonstrated associations between artistic training and interoceptive awareness and have not considered psychological variables in between them. In the preset study, we attempted to identify factors that may influence interoceptive awareness by comparing experts in the two domains of Noh performance with individuals who have no experience in Noh. Furthermore, we conducted exploratory analysis to examine the consistency with the dual-action hypothesis.

The purpose of this study was to examine changes in interoceptive awareness associated with the practice of Noh from multiple perspectives by surveying professional Noh performers and individual with no experience in Noh. Noh performance is composed of a series of basic movements called “kata (型),” and there seems to be relatively little variation in content across performers. This is another advantage of treating Noh as a subject for psychological research. Because Noh is performed by experts in several performance domains, we surveyed two groups of performers, shite-kata (actors; シテ方) and hayashi-kata (instrumentalists; 囃子方), to examine the differences between these domains. Actors play the role of the main character and engage in full-body movement. Whereas instrumentalists play the musical instruments and perform while seated. According to the dual-action hypothesis (Christensen et al., 2018), the elicitation and expression of physical states enhance interoceptive awareness. Therefore, it is possible that the training of actors, which actively uses broader areas of the body to express emotions, has a greater impact on interoceptive awareness than instrumentalists. Therefore, we hypothesized that levels of interoceptive awareness would be highest among actors, followed by musicians, and lowest among controls with no experience in Noh (Hypothesis 1). Based on previous findings demonstrating enhanced interoceptive accuracy among artists (Schirmer-Mokwa et al., 2015; Christensen et al., 2018) and on the idea that the dual-action in art training involves attention to body (Christensen et al., 2018), we expected that both accuracy-related and attention-related aspects of interoceptive awareness to be positively associated with Noh experience. Higher-order cognitive aspects, such as trust in bodily sensations, were examined exploratorily because no clear theoretical basis existed for generating specific predictions. Furthermore, if Noh practice contributes changes in interoceptive awareness, it is assumed that interoceptive awareness will increase as the amount of practice increases. Therefore, we hypothesized that indices such as years of Noh experience and amount of practice would be positively correlated with interoceptive awareness among Noh performers (Hypothesis 2). As in Hypothesis 1, positive associations with Noh experience were predicted for accuracy-related and attention-related variables, whereas higher-order aspects were examined exploratorily. To explore the mechanisms through which artistic practice may influence interoceptive awareness, two supplementary forms of data were collected. The first was a closed-ended psychological scale assessing the aspects of which participants were consciously aware during expressive activities (Takada et al., 2019). The second was open-ended questions. In the open-ended questions, “achieving a successful performance (舞台の成就)” was positioned as the central goal of Noh practice. Although “achieving a successful performance” is abstract and may be interpreted differently by individual performers, it generally refers to achieving a performance that the performer considers satisfactory. Participants were asked to describe what they kept in mind during the process of reaching this goal. Statements potentially related to interoception were subsequently extracted from these textual responses. Two groups of performers completed these supplementary questions after the scales regarding interoceptive awareness.

## 2 Methods

### 2.1 Participants

A questionnaire survey was conducted with a target number of 20 participants each for actors, instrumentalists, and controls with no Noh experience. In this study, performers were defined as “those who earn money from their activities as Noh performers.” Two professional Noh performers acquainted with the authors recruited the participants from their professional association and collected data in place of the authors. The controls were recruited through a crowdsourcing service (CrowdWorks; https://crowdworks.jp/).

To determine the sample size, we referred to a previous study (Christensen et al., 2018) which found an effect size of *f* = 0.77 using a one-way ANOVA (analysis of variance) of three groups (two experienced ballet dancers and non-practitioners). An effect size was calculated from the *F* value and degree of freedom. Assuming a one-way ANOVA as in the previous study, calculations were performed using GPower (Faul et al., 2007; version 3.1.9.7). It was estimated that a total of 30 participants was needed to detect the same level of effect as in the previous study, with a significance level of 0.05, and a power of 0.95. The target sample size was set larger than that in a previous study on ballet dancers (Christensen et al., 2018) to allow for a certain number of invalid responses due to this study being conducted using paper-based surveys, and the effect size may not be as large as in the previous study on ballet dancers (Christensen et al., 2018).

Sixteen actors and 14 instrumentalists responded to the survey. Controls were recruited under two criteria: (1) they reported no prior experience with Noh, and (2) the gender distribution of the control group was matched to that of the performers. Twenty people responded as the control group. After excluding data from those who failed to pass the attention check and whose data included a blank field, the final sample consisted of 10 actors (age: *M* = 45.60, *SD* = 12.29, range = 23–56), 12 instrumentalists (age: *M* = 47.50, *SD* = 10.85, range = 25–62), and 18 controls (age: *M* = 40.50, *SD* = 6.85, range = 31–52). All the participants included in the valid dataset were male. Although several females were included among the respondents, none remained after the missing data handling process. It should be noted that the attention-check item was included solely to identify insufficient-effort responses (see below) and is unrelated to the construct of “interoceptive attention.”

All the participants were paid 500 yen as a reward. The performers were paid an additional 1,000 yen as a reward for responding to the open-ended questions. The survey was approved by the Ethical Review Committee of the Graduate School of Education, Kyoto University (CPE-545) and conducted in accordance with the approved guidelines. Written or electrical informed consent was obtained from all participants.

### 2.2 Measures

Most of the scales used in this study are publicly available in each cited article; however, the modified versions used for the purposes of this study are available in the Supplementary File.

#### 2.2.1 Interoceptive Accuracy Scale (IAS)

The IAS measures the beliefs in how accurately an individual perceives interoception (Murphy et al., 2020). The Japanese version (Koike and Nomura, 2023) comprises 20 items in the form of “I can always accurately perceive when …,” with each of the 20 physical sensations inserted into “…” (e.g., I can always accurately perceive when my heart is beating fast). For each item, participants must choose one of the following options: (1) strongly disagree, (2) disagree, (3) neither agree nor disagree, (4) agree, or (5) strongly agree. Higher scores indicate stronger beliefs in interoceptive accuracy. Cronbach’s alpha coefficients were 0.91 and 0.89 in the Japanese validation study (Koike and Nomura, 2023) and this study, respectively. An attention-check item instructing participants to “select 1 for this item” was included in the questionnaire.

#### 2.2.2 Interoceptive Attention Scale (IATS)

The IATS measures beliefs about how much attention is paid to interoceptive signals (Gabriele et al., 2022). The Japanese version (Koike and Nomura, 2023) comprises 20 items as follows: “Most of the time my attention is focused on whether …,” with each of the physical sensations inserted into “…” (e.g., Most of the time my attention is focused on whether my heart is beating fast). For each item, participants must choose one of the following options: (1) strongly disagree, (2) disagree, (3) neither agree nor disagree, (4) agree, or (5) strongly agree. Higher scores indicate stronger beliefs in interoceptive attention. Cronbach’s alpha coefficients were 0.93 and 0.95 in the Japanese validation study (Koike and Nomura, 2023) and this study, respectively. An attention-check item instructing participants to “select 1 for this item” was included in the questionnaire.

#### 2.2.3 Multidimensional Assessment of Interoceptive Awareness (MAIA)

The MAIA measures multiple aspects of interoceptive awareness (Mehling et al., 2012). The IAS and IATS reflect relatively pure interoceptive awareness, such as accurate perception and attention, whereas the MAIA can examine relatively higher cognitive aspects that consider the relationships with emotion. The Japanese version (Shoji et al., 2018) consists of six subscales: attention regulation (e.g., I can return awareness to my body if I am distracted), body listening (e.g., I listen to my body to inform me about what to do), emotional awareness (e.g., I notice how my body changes when I feel happy/joyful), noticing (e.g., I notice how my body changes when I am angry), not distracting (e.g., I distract myself from sensations of discomfort), and trusting (e.g., I feel my body is a safe place). Participants must answer 25 items on a scale from (0) never true to (5) always true. Cronbach’ alpha coefficients of each subscale (attention regulation, body listening, emotional awareness, noticing, not distracting, and trusting) were 0.87, 0.84, 0.85, 0.74, 0.67, and 0.83 in the Japanese validation study, respectively (Shoji et al., 2018). These were 0.91, 0.84, 0.87, 0.75, 0.76, and 0.81 in this study, respectively.

#### 2.2.4 Ten item personality inventory

Several studies have reported that the personality traits of extraversion, neuroticism, and openness are correlated with interoception or art experience (Ivcevic et al., 2022; Pearson and Pfeifer, 2022). Items measuring extraversion (e.g., extraverted, enthusiastic), neuroticism (e.g., anxious, easily upset), and openness (e.g., open to new experiences, complex) from a Japanese version of the Ten Item Personality Inventory (Gosling et al., 2003; Oshio et al., 2012) were used to examine the relationships between these variables. The participants have to choose one of the following options: (1) strongly disagree, (2) moderately disagree, (3) slightly disagree, (4) neither agree nor disagree, (5) slightly agree, (6) moderately agree, or (7) strongly agree. Cronbach’ alpha coefficients were not reported in the Japanese validation study (Oshio et al., 2012) as each subscale contained only two items. However, in this study, the coefficients of each subscale (extraversion, neuroticism, openness) were 0.76, 0.54, and 0.60, respectively.

#### 2.2.5 Psychological scales for expressive awareness in musical performance

A scale measuring expressive awareness, that is, what the participants were conscious of during expressive activities, was used to explore the factors that can bring about changes in interoceptive awareness. The original version (Takada et al., 2019) was used for the instrumentalists. For actors, a modified version was used as this scale was originally developed for instrumental players. The items of modified version were described in the supplement. This scale consists of three subscales: conveying the message to the audience (e.g., I have something to convey to my listeners through my performance), matching the performer’s intention and method of expression (e.g., I can appropriately express my images and thoughts through my performance), and following the music score (e.g., The performer must perform while following the score). Participants answered 14 items on a scale from (1) strongly disagree to (5) strongly agree. In the development study, Cronbach’s alpha coefficients of each subscale (conveying, matching, following) were 0.83, 0.73, and 0.74, respectively. In this study, those in the actors version was 0.72, 0.60, and 0.79, and those in the instrumentalists (original) version were 0.80, 0.72, and 0.50, respectively.

#### 2.2.6 Open-ended items

Only the performers were asked to respond to the open-ended item. Four answer columns about “things I keep in mind toward achieving a successful performance” were provided: one for myself (mental and physical aspects) and one for others (mental and physical aspects). The participants were asked to respond intuitively and concretely to each column.

### 2.3 Procedure

The performers (actors and instrumentalists) were surveyed on paper, while the controls were surveyed online. Qualtrics, an online survey system (https://www.qualtrics.com/jp/), was used to collect data from the controls. First, all the participants answered questions on demographic variables (age, gender, and art experience: whether they were involved in artistic activities other than Noh). The performers indicated their role in Noh, years of experience, and hours of practice per week. The participants then answered a series of questionnaires. As the survey was conducted on paper, the order of presentation of the scales was fixed for all participants. The order of presentation was the same as that presented in the text above.

### 2.4 Data analysis

Statistical analyses of quantitative data were performed using R statistical analysis software (version 4.3.2; https://www.R-project.org/). The Shapiro–Wilk test was used to confirm the normality of the data. Eight scale scores regarding interoceptive awareness (IAS, IATS, and six subscales of MAIA) were included in primary analyses. For between-group comparisons, one-way ANOVA and *post-hoc t*-test were used for variables considered to follow a normal distribution. Non-parametric tests (Kruskal–Wallis test and *post-hoc* Wilcoxon rank-sum test) were used for variables that were not considered to follow a normal distribution. These analyses were performed using two-tailed tests, with a significance level of 5%. False discovery rate (FDR) correction using the Benjamini–Hochberg procedure was applied across the eight primary tests. When a significant main effect was observed, *post-hoc* comparisons were conducted using the Bonferroni correction (*α*: 0.050/3 = 0.0167). Corrected *p* values were reported. For effect size interpretation, partial eta squared (η_p_^2^) for ANOVA of 0.01, 0.06, and 0.14 were interpreted as small, medium, and large, respectively (Cohen, 1988). Cohen’s *d* for *t*-test of 0.20, 0.50, and 0.80 were interpreted small, medium, and large, respectively (Cohen, 1988). Eta squared based on the H-statistic (η^2^[H]) for Kruskal–Wallis test of 0.01, 0.06, and 0.14 were interpreted small, medium, and large, respectively (Tomczak and Tomczak, 2014). The *r* values for Wilcoxon rank-sum test of 0.10, 0.30, and 0.50 were interpreted small, medium, and large, respectively (Tomczak and Tomczak, 2014). Spearman’s rank correlation coefficient was calculated to examine the correlations between variables. FDR correction was also applied in the correlation tests across each correlation matrix. The *r* values as the correlation coefficient of 0.10, 0.30, and 0.50 were interpreted small, medium, and large, respectively (Cohen, 1988). Text data analysis was performed using KHcoder, a Japanese text analysis software package (Higuchi, 2016).

## 3 Results

The datasets generated and/or analyzed during the current study are available in the Open Science Framework at https://doi.org/10.17605/OSF.IO/EUBT6.

The mean and standard deviation of each variable were calculated for each group and are shown in Table 1, together with the results of the between-group comparison (as most participants did not have any art experience, excluding Noh, this variable was excluded from the analyses). Because a normal distribution was not assumed in the age (*W* = 0.72, *p* = 0.002), attention regulation (*W* = 0.83, *p* = 0.042), noticing (*W* = 0.84, *p* = 0.050), and not-distracting (*W* = 0.83, *p* = 0.035) of actors, and in the extraversion of controls (*W* = 0.85, *p* = 0.007), a non-parametric test was used for these variables.

**Table 1:** Mean value, SD, and results of comparison for each variable.

Variables	Actors	Instrumentalists	Controls	*F or H*	*p* (uncorrected)	*p* (corrected)	Eff size	Eff size (interpreted)
Age^k^	45.60 (12.29)	47.50 (10.85)	40.50 (6.85)	5.95	0.051		0.107	Medium
Years of art experience (except for Noh)	0.65 (1.60)	6.00 (13.14)		—	—		—	
Years of Noh experience	39.40 (12.89)	36.75 (12.81)	—	—	—		—	
Hours of Noh practice/week	19.50 (14.65)	14.67 (15.16)	—	—	—		—	
Interoceptive awareness								
IAS	3.95 (0.32)	3.60 (0.50)	3.90 (0.49)	1.94	0.158	0.158	0.095	Medium
IATS	3.41 (0.87)	2.73 (0.49)	2.38 (0.60)	8.13	0.001	**0.004**	0.305	Large
MAIA								
Attention regulation^k^	3.31 (0.66)	3.19 (0.83)	2.20 (0.74)	14.50	<0.001	**0.004**	0.337	Large
Body listening	3.05 (0.74)	2.63 (1.07)	1.96 (0.97)	4.54	0.017	**0.034**	0.197	Large
Emotional awareness	3.33 (1.15)	2.86 (1.17)	2.43 (1.03)	2.22	0.123	0.141	0.107	Medium
Noticing^k^	3.56 (0.83)	2.97 (1.04)	2.51 (0.61)	8.34	0.016	**0.034**	0.171	Large
Not-distracting^k^	2.37 (1.05)	1.83 (0.92)	2.59 (0.70)	5.48	0.065	0.086	0.094	Medium
Trusting	3.67 (0.79)	3.08 (1.24)	2.70 (0.80)	3.31	0.047	0.075	0.152	Large
Personality								
Extraversion^k^	3.35 (1.40)	3.29 (1.25)	2.83 (1.40)	1.81	0.404		0.005	Small
Neuroticism	4.90 (0.84)	4.04 (0.92)	4.11 (1.60)	1.58	0.219		0.079	Medium
Openness	3.90 (1.02)	4.13 (1.51)	3.56 (1.43)	0.65	0.528		0.034	Small
Expressive awareness								
Conveying	4.33 (0.76)	3.90 (0.76)	—	—	—		—	
Matching	2.94 (0.66)	3.12 (0.64)	—	—	—		—	
Following	2.68 (0.67)	2.82 (0.59)	—	—	—		—	

^K^Variables compared by Kruskal–Wallis test, values in (): standard deviation, boldface: *p* < 0.05, Eff size = η^2^_p_ for ANOVA; η^2^[H] for Kruskal–Wallis test, IAS, Interoceptive Accuracy Scale, IATS, Interoceptive Attention Scale, MAIA, Multidimensional Assessment of Interoceptive Awareness, Conveying, conveying the messages to the audience; Matching, Matching the performer’s intention and method for expression; Following, Following the music score.

### 3.1 Between-group comparison

Table 1 presents the results of the between-group comparisons. The main effect of group was not statistically significant in age and personality variables (extraversion, neuroticism, and openness). Given the low internal consistency of the personality variables, additional between-group comparisons were also conducted for each item. However, none of the six items showed significant differences (*F*s < 1.58 or *H*s < 4.18, *p*s > 0.124).

**Figure 1:**
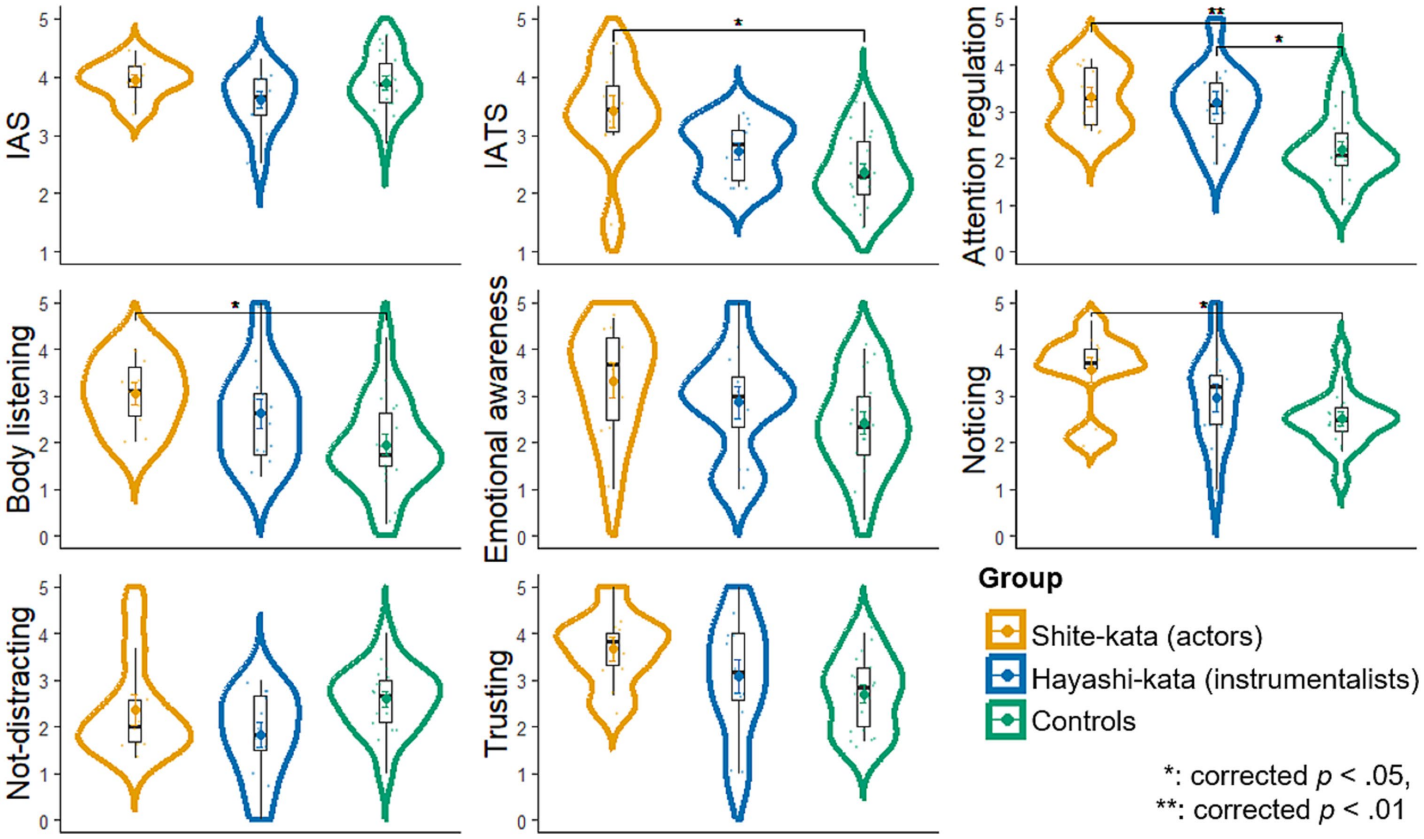
Scores of interoceptive awareness in each group. Large points, small points, and error bars indicate mean value, each participants’ data, and standard error, respectively. IAS, Interoceptive Accuracy Scale, IATS, Interoceptive Attention Scale.

The interoceptive awareness scores are shown in Figure 1. The main effect of group was not statistically significant in the score of IAS. The main effect of group was statistically significant in the score of IATS. *Post-hoc t*-test revealed that actors showed statistically significantly higher scores than the controls (*t*[13.9] = 3.34, *p* = 0.015, *d* = 1.38). The differences between the instrumentalists and the controls, and actors and instrumentalists were not statistically significant (*t*[26.7] = 1.77, *p* = 0.262, d = 0.65; *t*[13.6] = 2.19, *p* = 0.140, *d* = 0.96). The main effect of group was also statistically significant in attention regulation of the MAIA. *Post-hoc* Wilcoxon test revealed that actors showed statistically significantly higher scores than the controls (*W* = 160, *p* = 0.003, *r* = 0.63), as did the instrumentalists (*W* = 176, *p* = 0.012, *r* = 0.13). The difference between actors and instrumentalists was not statistically significant (*W* = 69, *p* = 1.00, *r* = 0.53). The main effect of group was statistically significant in body listening. *Post-hoc t*-test revealed that actors showed statistically significantly higher scores than controls (*t*[23.2] = 3.32, *p* = 0.009, *d* = 1.26). The differences between instrumentalists and the controls, and actors and instrumentalists were not statistically significant (*t*[19.4] = 1.09, *p* = 0.864, *d* = 0.46; *t*[22.1] = 1.73, *p* = 0.294, *d* = 0.65). The main effect of group was not statistically significant in emotional awareness. The main effect of group was statistically significant in noticing. *Post-hoc* Wilcoxon test revealed that actors showed statistically significantly higher scores than the controls (*W* = 146, *p* = 0.021, *r* = 0.51). The differences between instrumentalists and the controls, and actors and instrumentalists were not statistically significant (*W* = 140, *p* = 0.555, *r* = 0.25; *W* = 87, *p* = 0.237, *r* = 0.38). The main effect of group was not statistically significant in not-distracting. The main effect of group was not statistically significant in trusting.

### 3.2 Correlations with years of Noh experience

**Figure 2:**
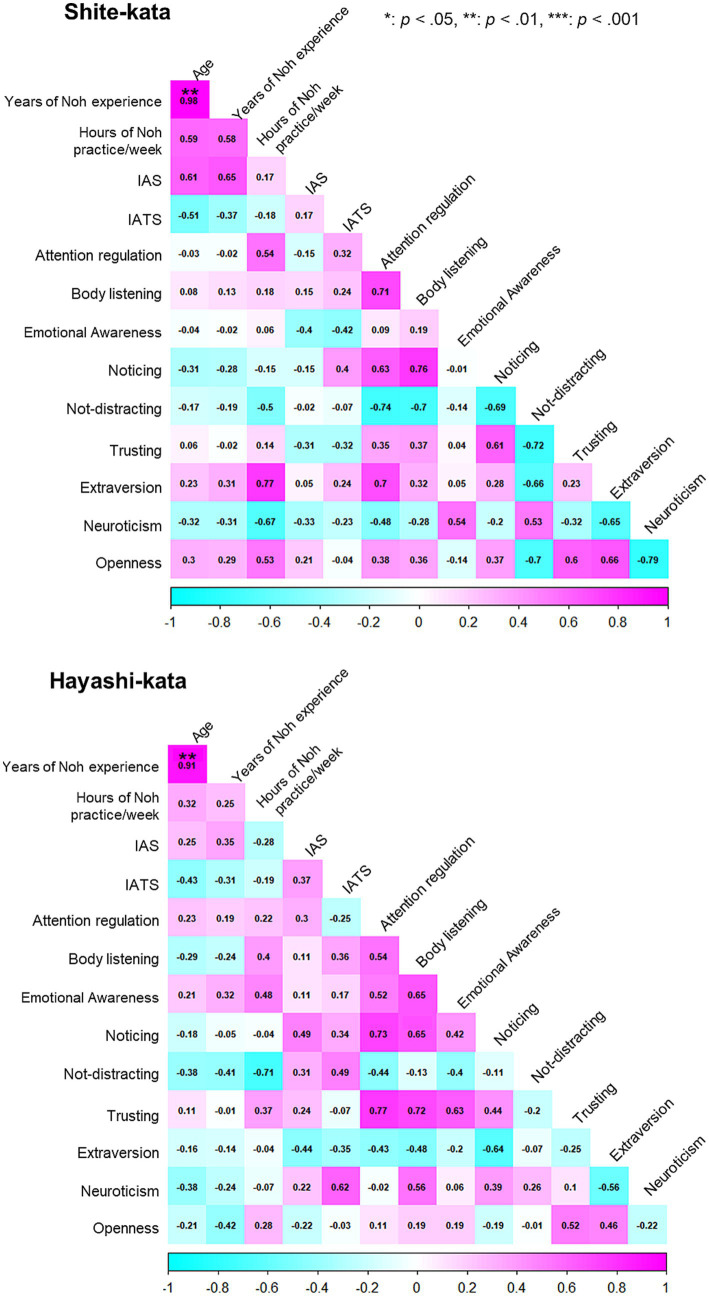
Correlations between each variable in performers. IAS, Interoceptive Accuracy Scale, IATS, Interoceptive Attention Scale.

A normal distribution was not assumed for the years of Noh experience in actors (*W* = 0.83, *p* = 0.038). Although hours of Noh practice is also the index of the amount of experience, we have not mentioned it and only shown it in the figures. The reason for this is that this index has low reliability because we did not distinguish “the time spent for their own practice” and “the time spent for supervision of their disciples.” The corrected *p* values are reported. To interpret the results for each group equivalently, rank correlation coefficients were calculated. Correlations between actors and instrumentalists are shown in Figure 2, and the controls are shown in Supplementary Figure S1.

There was a statistically significant strong positive correlation between years of experience and age (*r* = 0.98, *p* < 0.001, 95% CI [0.90, 0.99]) in actors. The correlation between years of Noh experience and IAS scores was also strong; however, it did not remain significant after FDR correction (*r* = 0.65, *p* = 0.235, 95% CI [0.04, 0.91]). Because age and years of experience were highly related among the actors, we conducted a partial correlation analysis controlling for age. This analysis also yielded a non-significant result (*r* = 0.32, *p* = 0.396). Moderate or weak correlations between years of experience and other scores of scales were not statistically significant (IATS: *r* = −0.37, *p* = 0.733, 95% CI [−0.81, 0.34]; attention regulation: *r* = −0.02, *p* = 0.970, 95% CI [−0.64, 0.61]; body listening: *r* = 0.13, *p* = 0.885, 95% CI [−0.55, 0.70]; emotional awareness: *r* = −0.02, *p* = 0.970, 95% CI [−0.64, 0.62]; noticing: *r* = −0.28, *p* = 0.740, 95% CI [−0.78, 0.42]; not-distracting: *r* = −0.19, *p* = 0.868, 95% CI [−0.73, 0.50]; trusting: *r* = −0.03, *p* = 0.970, 95% CI [−0.64, 0.62]; extraversion: *r* = 0.31, *p* = 0.736, 95% CI [−0.40, 0.78]; neuroticism: *r* = −0.31, *p* = 0.736, 95% CI [−79, 40]; openness: *r* = 0.29, *p* = 0.740, 95% CI [−0.42, 0.78]).

There was a strong positive correlation between years of experience and age in instrumentalists (*r* = 0.91, *p* < 0.001, 95% CI [0.71, 0.98]). Moderate or weak correlations between years of experience and other scales were not statistically significant (IAS: *r* = 0.35, *p* = 0.636, 95% CI [−0.28, 0.77]; IATS: *r* = −0.31, *p* = 0.689, 95% CI [−0.75, 0.32]; attention regulation: *r* = 0.19, *p* = 0.736, 95% CI [−0.43, 0.69]; body listening: *r* = −0.24, *p* = 0.736, 95% CI [−0.71, 0.39]; emotional awareness: *r* = 0.32, *p* = 0.689, 95% CI [−0.31, 0.75]; noticing: *r* = −0.05, *p* = 0.931, 95% CI [−0.61, 0.54]; not-distracting: *r* = −0.41, *p* = 0.604, 95% CI [−0.79, 0.22]; trusting: *r* = −0.01, *p* = 0.987, 95% CI [−0.58, 0.57]; extraversion: *r* = −0.14, *p* = 0.657, 95% CI [−0.66, 0.47]; neuroticism: *r* = −0.24, *p* = 0.447, 95% CI [−0.72, 38]; openness: *r* = −0.42, *p* = 0.178, 95% CI [−0.80, 0.21]).

### 3.3 Correlations between interoception and expressive awareness

**Figure 3:**
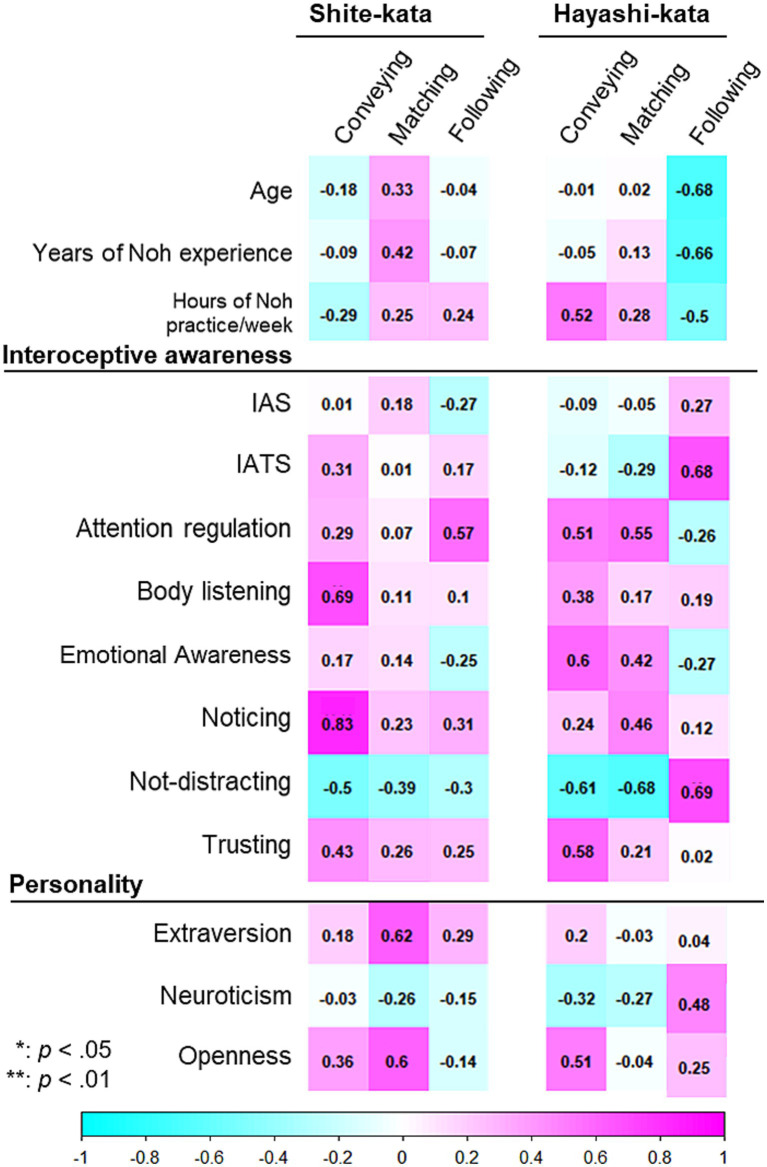
Correlations between expressive awareness and each variable. IAS, Interoceptive Accuracy Scale, IATS, Interoceptive Attention Scale.

To explore the factors that can bring about changes in interoceptive awareness, correlations between expressive awareness and each variable were calculated, as shown in Figure 3. In actors, although they did not remain significant after FDR correction, there were strong positive correlations between conveying and body listening (*r* = 0.69, *p* = 0.283, 95% CI [0.10, 0.92]), and between conveying and noticing (*r* = 0.83, *p* = 0.198, 95% CI [0.42, 0.96]). Moderate or weak correlations between expressive awareness and the other scales were not statistically significant (*r*s = 0.01–0.61, *p*s = 0.125–0.987; detailed statistics are shown in Supplementary Table S1).

In instrumentalists, although they did not remain significant after FDR correction, there werestrong positive correlations between conveying and emotional awareness (*r* = 0.60, *p* = 0.309, 95% CI [0.03, 0.87]), following and the score of IATS (*r* = 0.68, *p* = 0.238, 95% CI [0.17, 0.90]), and following and not-distracting (*r* = 0.69, *p* = 0.238, 95% CI [0.19, 0.90]). Moreover, there were statistically significant strong negative correlations between conveying and not-distracting (*r* = −0.61, *p* = 0.274, 95% CI [−0.88, −0.06]), matching and not-distracting (*r* = −0.68, *p* = 0.238, 95% CI [−0.90, −0.17]), following and age (*r* = −0.68, *p* = 0.238, 95% CI [−0.90, −0.18]), and following and years of experience (*r* = −0.66, *p* = 0.238, 95% CI [−0.89, −0.14]). Moderate or weak correlations between expressive awareness and the other scales were not statistically significant (*r*s = 0.01–0.60, *p*s = 0.238–0.987; detailed statistics are shown in Supplementary Table S1).

### 3.4 Text data from open-ended item

**Figure 4:**
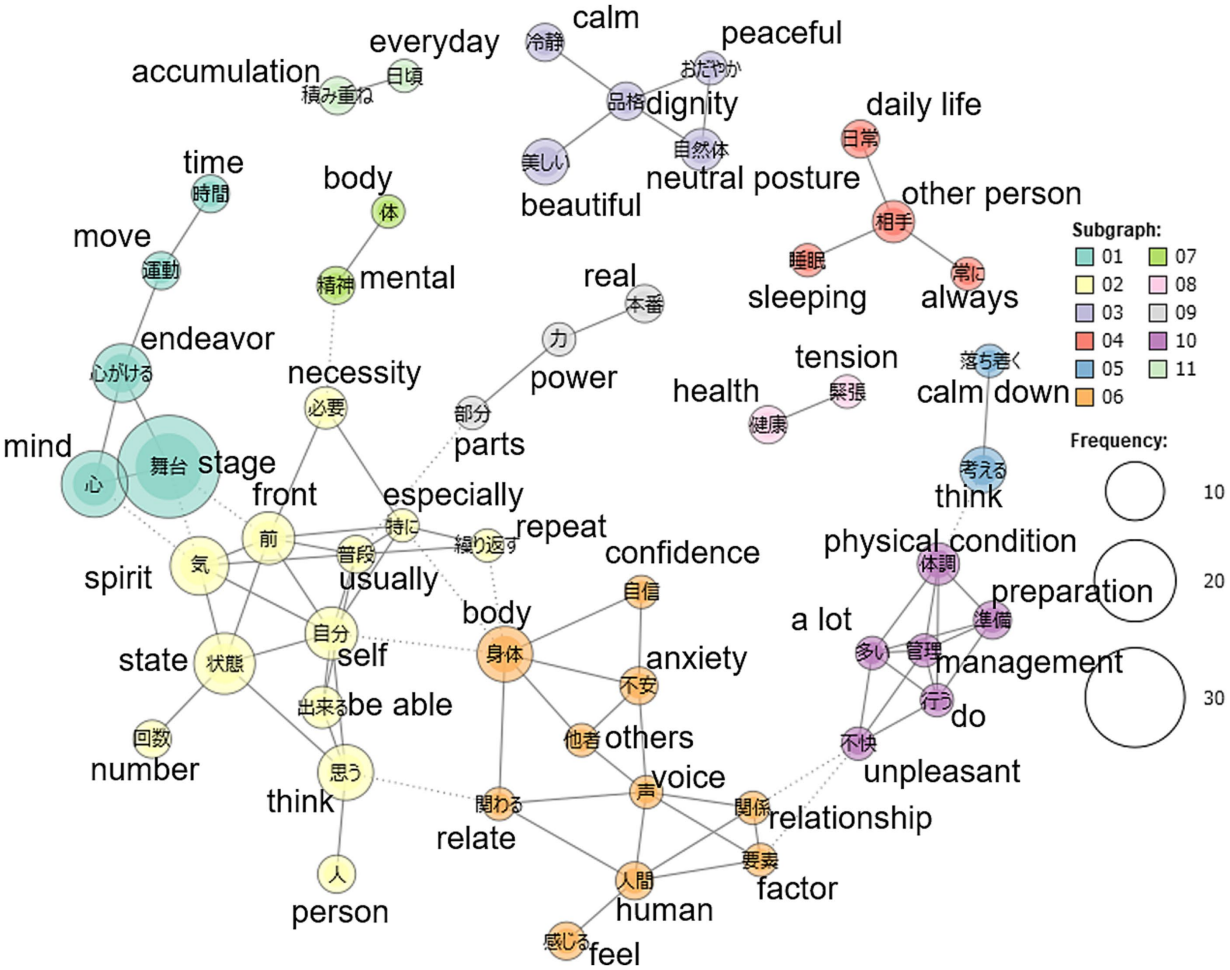
Co-occurrence network for the open-ended item of performers. Response for “What do you keep in mind for the fulfillment of the stage?”.

As there was relatively little text data collected, participants whose data had blank fields were combined, and the data of the four columns for “Things I keep in mind toward the fulfillment of the stage” were combined. Responses that did not contain meaningful content (e.g., “Nothing in particular”), or deviated from the theme of Noh practice (no participants met these criteria.) were excluded. A co-occurrence network based on the Jaccard index was constructed, with the minimum word frequency set at three (Figure 4). We conducted the same analysis for the data separated into groups (actors and instrumentalists), and the results are shown in Supplementary Figures S2-1, S2-2.

Co-occurrence network plots can be used to visualize groups of words that tend to co-occur as subgraphs. The authors subsequently interpret the themes represented by these word clusters. Although it was difficult to explain each subgraph theoretically, the words “repeat” and “accumulation” appeared repeatedly across some subgraphs (for example, “Practice hard,” “Repeat over and over”). This seems to indicate the importance of daily practice as a prerequisite for the fulfillment of the stage. Moreover, words related to emotional arousal, such as “calm” and “anxiety” also repeatedly appeared (for example, “Try to mentally calm down by considering various [about the audience and inside the dressing room on the day of the event] conditions”).

## 4 Discussion

This study aimed to examine the changes in interoceptive awareness associated with the practice of Noh. Three groups—actors, instrumentalists, and controls with no Noh experience—completed a battery of questionnaires assessing interoceptive awareness from multiple perspectives. Moreover, we attempted to identify factors may be associated with interoceptive awareness and used a scale for expressive awareness in musical performance and open-ended questions. The present findings should be interpreted as preliminary evidence suggesting that long-term Noh practice may be preferentially associated with attentional dimensions of interoceptive awareness.

In particular, actors, who perform with full-body movement, showed significantly higher scores for IATS, the MAIA subscales of Attention regulation, Body listening, and Noticing than the controls. Instrumentalists, who perform while seated, had significantly higher scores on attention regulation than the controls. Differences between actors and instrumentalists were not statistically significant. Thus, Hypothesis 1, that levels of interoceptive awareness would be highest among actors, followed by instrumentalists, and lowest among controls, was partially supported. Interestingly, this pattern was more evident for attention-related variables than for accuracy-related variables. IATS scores reflect a tendency to pay attention to interoceptive signals in most parts of daily life, and scores of several subscale in MAIA—attention regulation, body listening, and noticing—indicate a tendency to actively pay attention to interoceptive signals in specific contexts. Therefore, the influence of Noh experience was especially remarkable in terms of belief in interoceptive attention. Noh expertise might also be associated with higher-order cognitive aspects of interoception, such as trust in bodily sensations as measured by the Trusting subscale of the MAIA; however, this group difference did not remain significant after FDR correction. In contrast, years of Noh experience were not positively correlated with any of the variables regarding interoceptive awareness. Therefore, Hypothesis 2, which stated that indices such as years of Noh experience and amount of practice would be positively correlated with interoceptive awareness, was not supported. These results extended the findings of previous studies that artists have higher heartbeat perception performance to the belief aspect of interoceptive awareness (Schirmer-Mokwa et al., 2015; Christensen et al., 2018). Because neither group differences nor correlations with personality traits were not statistically significant, it seemed that there were no clear relationships in the context of the degree of Noh experience. Furthermore, exploratory analyses of expressive awareness and open-ended questions suggested a mechanism by which artistic training is associated with interoceptive awareness. Although the IATS score, belief to interoceptive attention was higher among performers, it did not show a significant correlation with years of experience. In contrast, the between-group difference in IAS score, belief to interoceptive accuracy was not statistically significant. One possible factor is that interoceptive attention is more sensitive to Noh practice than is accuracy. Most performers in this study were proficient, with more than 20 years of experience. It is possible that at the 20-year mark, the effect of practice on interoceptive attention had already reached its maximum, and that the correlation confirmed by the ceiling effect was small. By contrast, belief about accuracy may still be developing even among experts, and more experience is needed to improve it. The association did not survive FDR correction, although the effect size regarding correlation between years of experience and IAS scores among in actors remained relatively large (*r* = 0.65). Given that the present study was intended as a preliminary investigation of the associations between expertise and multiple interoceptive variables, and considering the magnitude of this effect, mentioning this finding is still thought to have some value. The development of beliefs about accuracy may require the experience of both the physical sensations occurring (and paying attention to them) and the results of actions based on these physical sensations, which may have made it relatively difficult for the effects of practice to manifest themselves. This view is supported by the fact that the belief in attention is more malleable than the belief in accuracy (Koike and Nomura, 2023). Another possibility is that this pattern reflects characteristics specific to Noh training. Noh, especially the performance of shite-kata (actors), is characterized by emotional expression conveyed exquisitely within relatively static basic movements, rather than by direct emotional expression. Long-term practice involving sustained attention to breathing and bodily balance may therefore be more closely associated with attention-related aspects of interoception. Regardless of the underlying explanation, the interesting results regarding the different patterns of association between interoceptive awareness and Noh practice by scale suggest that Noh practice may have different effects on different aspects of interoception. This possibility can be further verified by examining the growth curve of each scale from the time before Noh performance is begun to the time when the individual becomes a proficient Noh performer.

The results of exploratory analyses were also broadly consistent with the dual-action hypothesis (Christensen et al., 2018). Although this interpretation should be treated with caution because it is based on effect sizes rather than statistical significance, the newly suggested relationships with conveying subscale in the scale regarding expressive awareness adds further explanatory power to the dual-action hypothesis. Simultaneously, the results of the text data analysis suggest that there are other mechanisms by which the practice of Noh can enhance interoceptive awareness than those postulated in the dual-action hypothesis. For example, interoceptive awareness is particularly related to the perception of arousal among emotions (Barrett et al., 2004; Suzuki and Yamamoto, 2023), and the frequent occurrence of arousal-related words in performers’ descriptions suggests that active attention to the body to control arousal may also contribute to the long-term improvement of interoceptive awareness.

Although this study provides new insights into artistic activity and interoception, it has several limitations. First, it addressed several aspects of interoceptive awareness, such as belief on accuracy and attention, all of which are subjective measures. Research using behavioral measures has found higher interoceptive awareness regardless of the form of art activity (Schirmer-Mokwa et al., 2015; Christensen et al., 2018), possibly because behavioral measures are more transformative than subjective measures (Ferentzi et al., 2018). Future studies should be comprehensive, using behavioral (Schandry, 1981) and physiological indices (Coll et al., 2021) of interoception. Secondly, the sample size was relatively small though the target sample size was set larger than that in a previous study on ballet dancers (Christensen et al., 2018). Even when statistically significant effects were observed, the precision of the effect size estimates remained limited, as reflected in the wide confidence intervals. Therefore, the reported effect sizes should be interpreted as preliminary estimates rather than precise estimates of population parameters. Furthermore, the subgraphs in the co-occurrence network diagram may only reflect the data for each individual. For a more reliable interpretation, it is necessary to examine a larger sample size based on the results of this study. A larger sample size would also make it possible to fit a more complex model, such as growth curve of interoceptive awareness. Third, the IAS, IATS, and MAIA used in this study are only measures of interoceptive awareness in daily life. It is significant that this study showed that the practice of Noh is associated with the interoceptive awareness in daily life. However, if interoceptive awareness in limited situations, such as during a performance, can be measured, it would be possible to gain more insight into the psychological and physical techniques of Noh performers. Fourth, several methodological issues arising from the limited accessibility of the sample should be acknowledged. Data were collected using different response formats: paper-based questionnaires for the Noh performers and online questionnaires for the control participants. Potential differences between the groups in motivation, response style, or survey context were not controlled for and may have influenced the results. In addition, the questionnaire order was fixed, which may have affected participants’ responses. Although no evidence currently suggests that response format or questionnaire order substantially influences interoceptive measures, future studies should attempt to minimize these potential sources of bias whenever possible. Furthermore, all participants in the present study were male. Previous research has reported gender differences in several interoceptive variables; for example, males tend to exhibit higher heartbeat perception accuracy, whereas females tend to score higher on the Noticing subscale of the MAIA (Grabauskaitė et al., 2017). Such differences may reflect not only biological factors, such as variations in heartbeat intensity (Knapp-Kline and Kline, 2005), but also cognitive and sociocultural factors, including health literacy (Whitaker et al., 2015). Although the present study complements previous findings obtained from predominantly female samples (Christensen et al., 2018), caution is warranted when generalizing the findings to broader populations.

This study examined the relationship between Noh practice and interoceptive awareness using a set of scales that measured interoceptive awareness from multiple perspectives. The results of the study showed that actors who perform with full-body movement were more likely to report greater attention to interoceptive signals. Furthermore, an exploratory analysis provided preliminary insights into potential mechanism linking Noh experience and interoceptive awareness. Future research should examine the effects of the practice of Noh using behavioral and physiological indices of interoception and construct a new experimental paradigm based on the present exploratory findings. Such work may yield a deeper understanding of the psychological and physical techniques cultivated through Noh performers and their potential applications.

## Data Availability

The datasets presented in this study can be found in online repositories. The names of the repository/repositories and accession number(s) can be found at: https://doi.org/10.17605/OSF.IO/EUBT6.
