# Supplementary material for: Improved interoceptive awareness in professional performers of the traditional Japanese theatrical art “Noh”
Source: Front Psychol. 2026 Jul 13;17:1818785. doi: 10.3389/fpsyg.2026.1818785 (PMC13402160; doi:10.3389/fpsyg.2026.1818785)
Supplement: Supplementary file 1 [file Supplementary_file_1.docx]

Supplementary Material

# Supplementary Figures and Tables

**Figure S1. Correlations between each variable in Controls.** IAS = Interoceptive Accuracy Scale, IATS = Interoceptive Attention Scale.


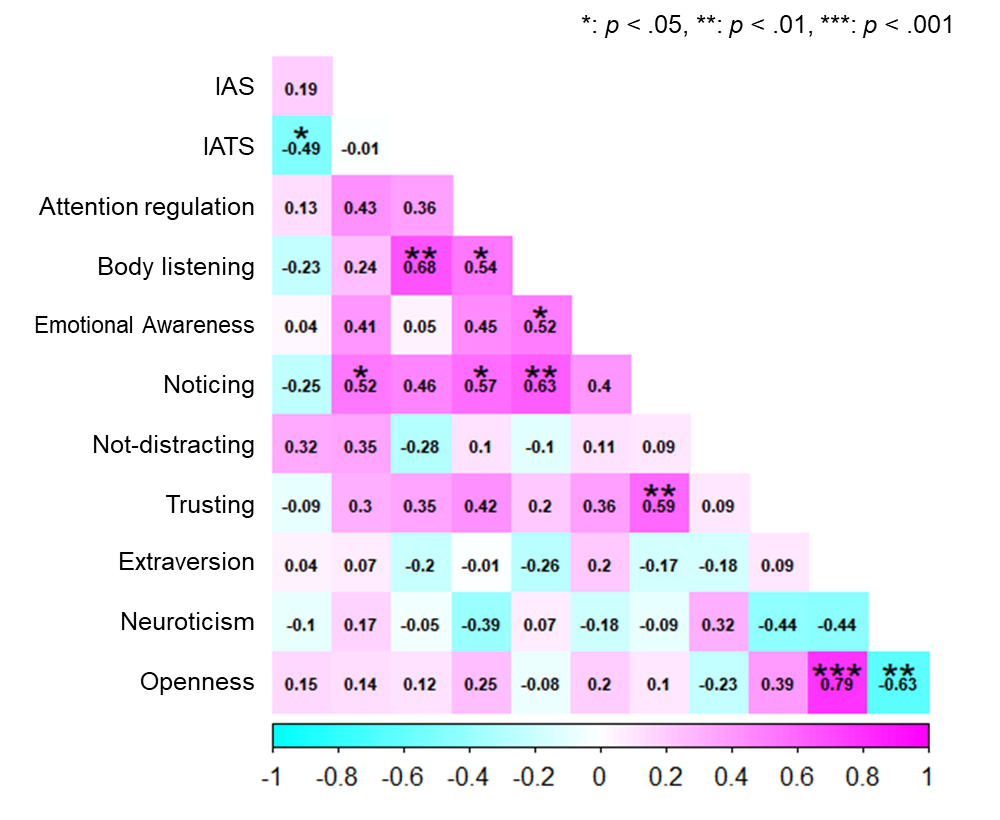


**Figure S2-1. Co-occurrence network for open-ended item of Shite-kata.** Response for “What do you keep in mind for the fulfillment of the stage?”.


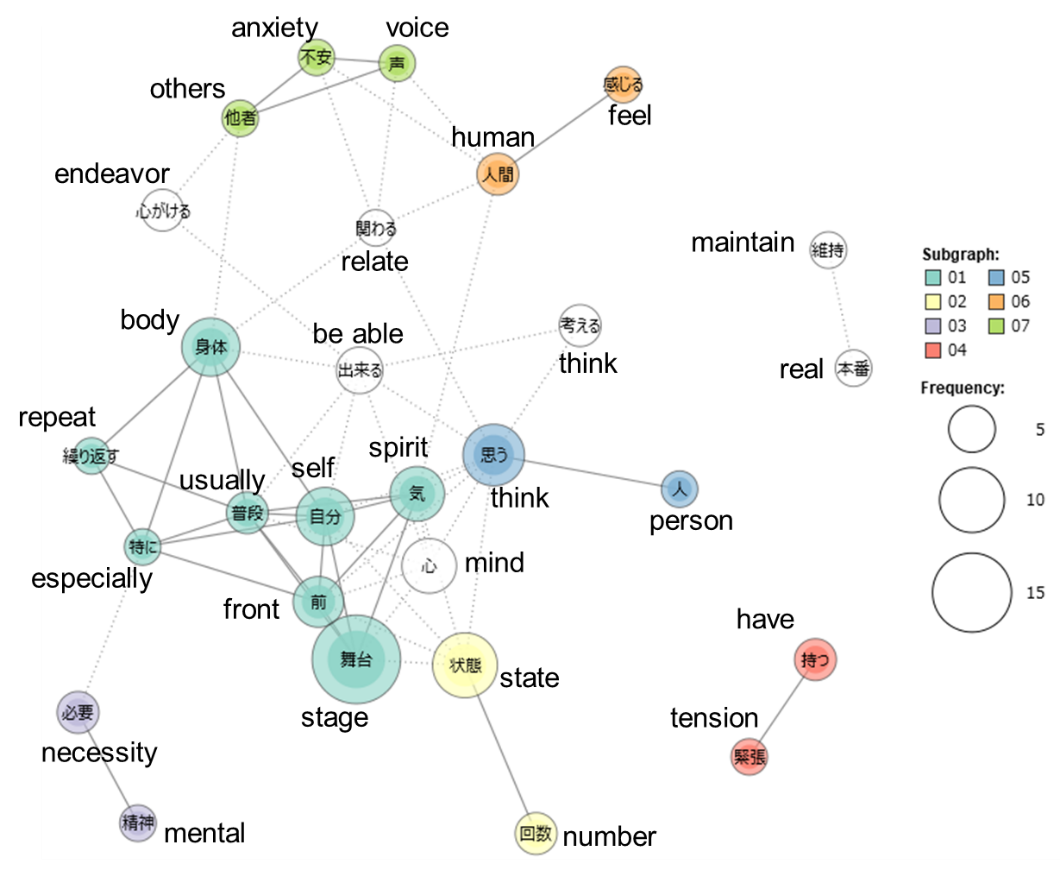


**Figure S2-2. Co-occurrence network for open-ended item of Hayashi-kata.** Response for “What do you keep in mind for the fulfillment of the stage?”.


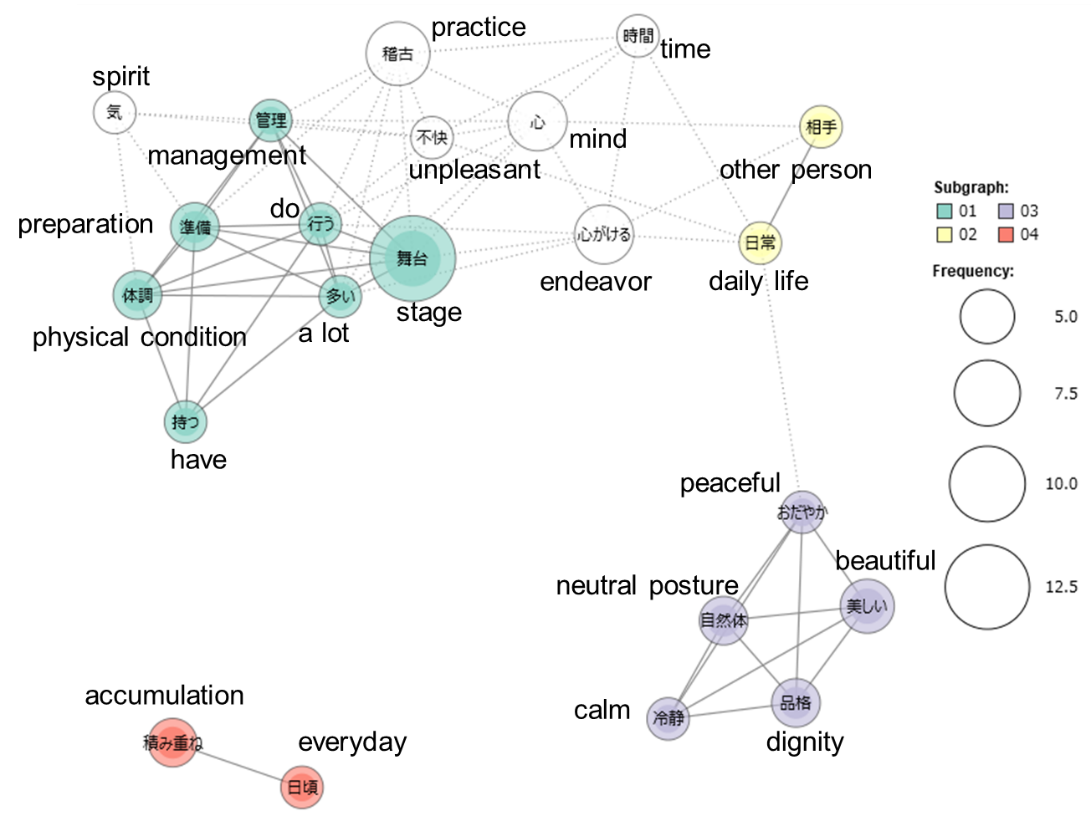


**Table S1. Correlations between expressive awareness and variables.** Corrected *p* values were reported.

|  | **Shite-kata (actors)** | | |  |  |  |  |  |  |
| --- | --- | --- | --- | --- | --- | --- | --- | --- | --- |
|  | Conveying | | | Matching | | | Following | | |
|  | *r* | *p* | 95% CI | *r* | *p* | 95% CI | *r* | *p* | 95% CI |
| Age | -.18 | .873 | [-.73, .51] | .33 | .826 | [-.38, .79] | -.04 | .981 | [-.66, .60] |
| Years of Noh experiences | -.09 | .959 | [-.68, .57] | .42 | .796 | [-.29, .83] | -.07 | .968 | [-.67, .58] |
| Hours of Noh practice/week | -.29 | .826 | [-.78, .41] | .25 | .849 | [-.44, .76] | .24 | .849 | [-.46, .76] |
| **Interoceptive awareness** |  |  |  |  |  |  |  |  |  |
| IAS | .01 | .987 | [-.62, .64] | .18 | .873 | [-.50, .73] | -.27 | .849 | [-.77, .43] |
| IATS | .31 | .826 | [-.40, .79] | .01 | .987 | [-.63, .63] | .17 | .873 | [-.51, .72] |
| MAIA |  |  |  |  |  |  |  |  |  |
| Attention regulation | .29 | .826 | [-.42, .78] | .07 | .968 | [-.58, .67] | .57 | .416 | [-.09, .88] |
| Body listening | .69 | .283 | [.11, .92] | .11 | .924 | [-.56, .69] | .10 | .930 | [-.56, .69] |
| Emotional awareness | .17 | .873 | [-.52, .72] | .13 | .873 | [-.54, .71] | -.25 | .849 | [-.76, .45] |
| Noticing | .83 | .198 | [.42, .96] | .23 | .849 | [-.47, .75] | .31 | .826 | [-.39, .79] |
| Not-distracting | -.50 | .547 | [-.27, .83] | -.39 | .826 | [-.82, .32] | -.30 | .826 | [-.78, .41] |
| Trusting | .43 | .792 | [-.27, .83] | .26 | .849 | [-.44, .77] | .37 | .849 | [-.45, .76] |
| **Personality** |  |  |  |  |  |  |  |  |  |
| Extraversion | .18 | .873 | [-.50, .73] | .62 | .365 | [-.01, .90] | .29 | .826 | [-.42, .78] |
| Neuroticism | -.03 | .981 | [-.64, .61] | -.26 | .849 | [-.77, .44] | -.15 | .873 | [-.71, .53] |
| Openness | .36 | .826 | [-.35, .81] | .60 | .365 | [-.04, .89] | -.14 | .873 | [-.71, .53] |
|  |  |  |  |  |  |  |  |  |  |
|  | **Hayashi-kata (instrumentalists)** | | | |  |  |  |  |  |
|  | Conveying | | | Matching | | | Following | | |
|  | *r* | *p* | 95% CI | *r* | *p* | 95% CI | *r* | *p* | 95% CI |
| Age | -.01 | .987 | [-.58, .57] | -.02 | .982 | [-.56, .59] | -.68 | .238 | [-.90, -.18] |
| Years of Noh experiences | -.05 | .963 | [-.60, .54] | .13 | .862 | [-.48, .66] | -.66 | .238 | [-.89, -.14] |
| Hours of Noh practice/week | .52 | .436 | [-.07, .84] | .28 | .749 | [-.35, .74] | -.50 | .450 | [-.83, .11] |
| **Interoceptive awareness** |  |  |  |  |  |  |  |  |  |
| IAS | -.09 | .909 | [-.63, .51] | -.05 | .963 | [-.61, .54] | .27 | .749 | [-.36, .73] |
| IATS | -.12 | .879 | [-.63, .51] | -.29 | .749 | [-.74, .34] | .68 | .238 | [.17, .90] |
| MAIA |  |  |  |  |  |  |  |  |  |
| Attention regulation | .51 | .436 | [-.09, .84] | .55 | .396 | [-.04, .85] | -.26 | .749 | [-.73, .37] |
| Body listening | .38 | .581 | [-.24, .78] | .17 | .791 | [-.45, .68] | .19 | .762 | [-.43, .69] |
| Emotional awareness | .60 | .309 | [.03, .87] | .42 | .540 | [-.20, .80] | -.27 | .749 | [-.73, .36] |
| Noticing | .24 | .749 | [-.39, .71] | .46 | .482 | [-.15, .82] | .12 | .879 | [-.49, .65] |
| Not-distracting | -.61 | .274 | [-.88, -.06] | -.68 | .238 | [-.90, -.17] | .69 | .238 | [.19, .90] |
| Trusting | .58 | .331 | [.02, .87] | .21 | .756 | [-.41, .70] | .02 | .982 | [-.56, .58] |
| **Personality** |  |  |  |  |  |  |  |  |  |
| Extraversion | .20 | .762 | [-.42, .69] | -.03 | .963 | [-.60, .55] | .04 | .963 | [-.54, .60] |
| Neuroticism | -.32 | .694 | [-.76, .31] | -.27 | .749 | [-.73, .36] | .48 | .459 | [-.14, .82] |
| Openness | .51 | .436 | [-.09, .84] | -.04 | .963 | [-.60, .55] | .25 | .749 | [-.38, .72] |
| Boldface: *p* < .05, IAS = Interoceptive Accuracy Scale, IATS = Interoceptive Attention Scale, MAIA = Multidimensional Assessment of Interoceptive Awareness, Conveying = Conveying the messages to the audience, Matching = Matching the performer’s intention and method for expression, Following = Following the music score. | | | | | | | | | |

# Other Supplementary information

**Psychological Scales for Expressive awareness in Musical performance for actors (shite-kata).** Items were modified from Takada et al. (2019). The provisional English version was prepared by the author.

**Terms.** Fushi-zuke: musical symbols, Goma: musical notes

**Instruction and items.**演能に対するあなたのお考えについてお聞きします。それぞれ，当てはまるもの一つに〇をつけてください。
We would like to ask you a few questions about your opinions on the performance of Noh. Please tick the one that applies to you.
1. 私は観客に何らかのメッセージを伝えるために演技する方だ。
 I am the one who performs to convey some message to the audience.
2. 私は，謡本や片付けに書かれた要素（節付けやゴマなど）を音や動きに出せるが， どのように表現していいのか分からないことがある。
 I can produce the elements (e.g. fushi-zuke and goma) written in scores in sound and movement, but sometimes I do not know how to express them.
3. 私は自分の舞台を通じて，観客に伝えたいことがある。
 I have something I want to convey the audience through my performance.
4. 私には，舞台を通じて観客に伝えたいことはあまりない。
 I don't have much that I want to convey to the audience through my performance.
5. 私にとって，自分のイメージを適切に表現するよりも，謡本や片付けに忠実であることの方が大切だ。
 It is more important for me to be faithful to scores than to express my image properly.
6. 私は表現したい内容があるが，それを表現する方法が分からない。
 I have something I want to express, but I don't know how to express it.
7. 私は自分のイメージや考えを舞台を通して適切に表現できる。
 I can express my images and ideas appropriately on the stage.
8. 私は，舞台を通じて観客に何らかのメッセージを伝えることは大切だと思う。
 I think it is important to convey some kind of message to the audience through the stage.
9. 私は自分が表現したいことにぴったりとくる方法を用いて演技する方だ。
 I perform in a way that fits what I want to express.
10. 私は観客にイメージを伝えるテクニックを持っている。
 I have techniques to convey images to the audience.
11. 演者は，謡本や片付けに書かれていない要素（節付けやゴマなど）を音や動きに出してはならない。
 Performers must not produce elements in sound or movement that are not written down in scores (e.g. fushi-zuke and goma).
12. 楽曲の解釈には，謡本や片付けに書かれている要素（節付けやゴマなど）が最も重要だ。
 The elements written in scores (e.g. fushi-zuke and goma) are the most important for the interpretation of the piece.
13. 演者は謡本や片付けに忠実に演技しなければならない。
 Performers must perform faithfully to scores.
14. よい舞台とは，謡本や片付けに忠実であることだと思う。
 I think that a good performance is faithful to scores.
